# Sustainable Medical Teaching and Learning During the COVID-19 Pandemic: Surviving the New Normal

**DOI:** 10.21315/mjms2020.27.3.14

**Published:** 2020-06-30

**Authors:** Muhamad Saiful Bahri Yusoff, Siti Nurma Hanim Hadie, Irfan Mohamad, Nani Draman, Ismail Muhd Al-Aarifin, Wan Faiziah Wan Abdul Rahman, Mohamad Najib Mat Pa, Nor Azwany Yaacob

**Affiliations:** 1 Department of Medical Education, School of Medical Sciences, Universiti Sains Malaysia, Kubang Kerian, Kelantan, Malaysia; 2 Department of Anatomy, School of Medical Sciences, Universiti Sains Malaysia, Kubang Kerian, Kelantan, Malaysia; 3 Department of Otorhinolaryngology-Head & Neck Surgery, School of Medical Sciences, Universiti Sains Malaysia, Kubang Kerian, Kelantan, Malaysia; 4 Department of Family Medicine, School of Medical Sciences, Universiti Sains Malaysia, Kubang Kerian, Kelantan, Malaysia; 5 Department of Pathology, School of Medical Sciences, Universiti Sains Malaysia, Kubang Kerian, Kelantan, Malaysia; 6 Department of Community Medicine, School of Medical Sciences, Universiti Sains Malaysia, Kubang Kerian, Kelantan, Malaysia

**Keywords:** medical education, teaching, learning, distance education, COVID-19

## Abstract

During the first phase of the Movement Control Order, many medical lecturers had difficulty adapting to the online teaching and learning methods that were made compulsory by the institutional directives. Some of these lecturers are clinicians who need to juggle between clinical work and teaching, and consider a two-week adaptation during this period to be not enough. Furthermore, converting traditional face-to-face learning to online formats for undergraduate and postgraduate clinical programmes would reduce the learning outcomes, especially those related to clinical applications and the acquisition of new skills. This editorial discusses the impact that movement restrictions have had on medical teaching and learning, the alternatives and challenges and the way forward.

## The Clicking Time Bomb

The coronavirus disease 2019 (COVID-19) outbreak was designated as a public health emergency on 30 January 2020 (1). Subsequently, the World Health Organization (WHO) declared COVID-19 to be a pandemic after 200,000 cases had been detected with 8,000 deaths across 160 countries (2). The rapid spread of COVID-19 has had an enormous impact on many aspects of human life including social development and education and has caused many countries to activate emergency risk management (3).

**Figure 1:**
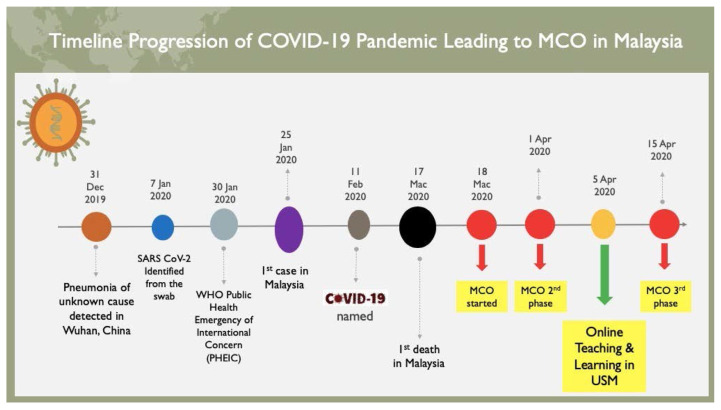
Chronological events during COVID-19 pandemic leading to the MCO and USM online teaching and learning initiatives

Malaysia reported its first COVID-19 case on 25 January 2020; the patient contracted the disease from Chinese tourists who had crossed the border from Singapore two days earlier. After that, the number of cases continued to escalate, few clusters of the spread could be identified and the first death was reported on 17 March 2020 (4). Malaysia took preventive action with the announcement of the Movement Control Order (MCO) on 16 March 2020 (5), through the Prevention and Control of Infectious Diseases Act 1988 and the Police Act 1967 (Figure 1). The first phase came into effect on 18 March 2020, with extensions to be announced as per Ministry of Health inputs and the implementation managed by the Malaysian National Security Council.

## The Dawn of the MCO

The main objective of the MCO is to isolate the sources of COVID-19 cases and break the chain of infection. The methods of preventing the disease and isolating the sources of the cases include prohibition of movement and mass assemblies nationwide, including all religious, social, sports, cultural and education activities (6). However, the MCO that resulted from the COVID-19 pandemic has created significant challenges for the higher education community in ensuring continuous provision of education to the students.

In terms of undergraduate and postgraduate medical curricula, the continuity of medical programmes is important so that medical students can become familiar with practice settings and the teams to which they are assigned. At the same time, it is important for teachers of medicine to assess their students’ abilities with regard to patient care and safety, as well as the development of their professional behaviours. Continuity in the relationships between teachers and students in medicine also allows the teachers to evaluate their students’ learning needs, not only those related to cognitive competency but also their requirements in addressing other issues relating to mastering skills and acquiring learning values (7). It should be noted that if the duration for the MCO following the COVID-19 outbreak were to exceed two months, which is now the case, it was essential for medical schools to activate emergency risk management for the implementation of online teaching and learning during the outbreak.

## From Room to Roam

Online teaching and learning have rapidly become a delivery method for higher education in many countries including Malaysia. The new technologies appear to offer many advantages over conventional formats, such as being more cost effective, giving greater levels of access to students, providing more flexible teaching and learning approaches and, therefore, enhancing educational opportunities (8). Since their introduction, online teaching and learning have often been regarded as a supplementary teaching approach rather than replacing face-to face classes (9). Online sessions were initially adopted to overcome problems with traditional face-to-face teaching, such as to improve opportunities for students to access formal education, to increase students’ autonomy and expedite the completion of their degree programmes (10) and, hence, to enable the rapid growth of blended learning (11).

However, online teaching continues to be an adjunct teaching method for medical curricula, as face-to face teaching is still required for the development of certain learning outcomes such as the acquisition of clinical skills and development of values (i.e. professionalism) (12). In spite of the increasing use of technology-enhanced learning in medical curricula (13, 14), it was never viewed as a method that would totally replace face-to-face teaching until the COVID-19 outbreak. Being able to conduct full-blown online teaching and learning in medical schools during this pandemic is critical to ensure the continuity of educational delivery to medical students after the prohibition of movement and mass assemblies had been undertaken to break the chain of infection.

## The Challenges are Real

Nevertheless, the adoption of online learning is not without obstacles. Studies have shown that the attitudes of teachers and learners are strong determinants of the successful implementation of online learning and of its effectiveness (15–17). The interest and curiosity of teachers have been the main impetus for trying the online learning mode. The greatest resistance to it is found in relation to logistical and organisational problems, mainly resulting from lack of access to the internet or not having adequate skills in using it, and in cultural difficulties, such as strong preferences for face-to-face interaction. There have also been misconceptions about distance and online teaching methods that have resulted in lecturers being opposed to conducting online learning. Among these are lecturers and students’ perceptions that they have total freedom with regard to participation in time and in space (18). However, collaborative learning processes in fact require mutual commitment, whether they are conducted in real-time or not (15). There is also frequent disagreement regarding the potential of online written and asynchronous communication, with reference to their abilities to foster deep learning (15). Some other misconceptions and anxieties that lecturers have about online teaching are that it lacks digital literacy or that using computers could not allow for the achievement of learning outcomes in their programmes. Some lecturers insist that students love face-to-face teaching, even though there is no empirical or proven evidence for this (18, 19).

## The Way Forward

A number of lecturers have echoed their concerns about converting face-to-face learning to online formats for undergraduate and postgraduate clinical programmes, which they believe might limit their chances of achieving their learning outcomes, especially those related to clinical application and the acquisition of skills. A number of suggestions have been made to overcome the teacher’s resistance and misconceptions regarding online teaching and learning (20, 21). Successful online teaching is multifactorial, as it is the result of complex interplay among personal, pedagogical, contextual and organisational factors within higher education institutions. These factors include providing lecturers with continuous professional development training pertaining to the online teaching and learning, providing good internet support and web-based platforms for the lecturers so that they can develop their own autonomies and creating positive cultural environments such as rewards and recognition with regard to online education (21). These efforts would be able to stimulate the internal motivation and willingness of the lecturers to conduct online teaching as one of their routine teaching approaches.

**Figure 2:**
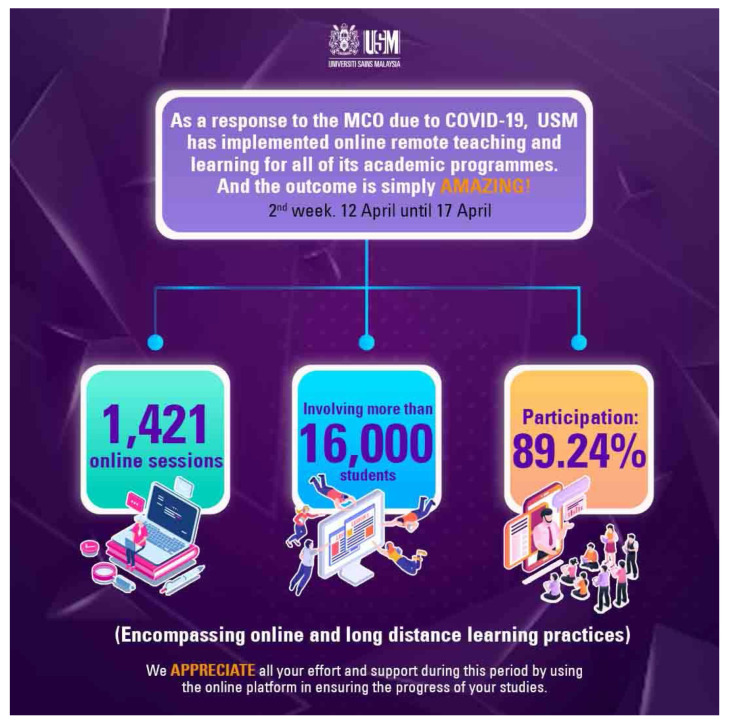
USM online teaching and learning sessions conducted within its 2nd week (12 April–17 April 2020)

Clearly, the unprecedented outbreak of the COVID-19 pandemic has led to changes in the way online teaching and learning are perceived. With the complete lockdowns, called the MCO in Malaysia, that were enforced in many countries, educators worldwide have been left with no options except to conduct online teaching and learning to ensure continuous provision of education to students. This has also been the unique real-time experience of many medical lecturers in Malaysian medical schools, as they must conduct online teaching and learning within their own capabilities and in spite of their limitations. At the time of writing this, Universiti Sains Malaysia has just released an appreciation note on the amazing number of 1,421 online teaching and learning sessions conducted in the second week (12 April–17 April 2020) of its implementation directive (Figure 2) (22). A study is needed to provide an initial evaluation of the implementation of online teaching, learning and assessment during the MCO, by exploring the enablers and the challenges faced by the lecturers.
